# Is Black Carbon an Unimportant Ice‐Nucleating Particle in Mixed‐Phase Clouds?

**DOI:** 10.1002/2017JD027831

**Published:** 2018-04-26

**Authors:** Jesús Vergara‐Temprado, Mark A. Holden, Thomas R. Orton, Daniel O'Sullivan, Nsikanabasi S. Umo, Jo Browse, Carly Reddington, María Teresa Baeza‐Romero, Jenny M. Jones, Amanda Lea‐Langton, Alan Williams, Ken S. Carslaw, Benjamin J. Murray

**Affiliations:** ^1^ Institute for Climate and Atmospheric Science, School of Earth and Environment University of Leeds Leeds UK; ^2^ Now at Institute for Atmospheric and Climate Science ETH Zurich Zurich Switzerland; ^3^ School of Chemistry University of Leeds Leeds UK; ^4^ Now at Lloyd's of London London UK; ^5^ Now at Institute for Meteorology and Climate Research‐Atmospheric Aerosol Research Karlsruhe Institute of Technology Eggenstein‐Leopoldshafen Germany; ^6^ Now at School of Geography University of Exeter Penryn Cornwall UK; ^7^ Escuela de Ingeniería Industrial de Toledo Universidad de Castilla‐La Mancha Toledo Spain; ^8^ School of Chemical and Process Engineering University of Leeds Leeds UK; ^9^ Now at School of Mechanical, Aerospace and Civil Engineering University of Manchester Manchester UK

**Keywords:** black carbon, ice nucleation, aerosol, global modeling, mixed‐phase clouds, clouds

## Abstract

It has been hypothesized that black carbon (BC) influences mixed‐phase clouds by acting as an ice‐nucleating particle (INP). However, the literature data for ice nucleation by BC immersed in supercooled water are extremely varied, with some studies reporting that BC is very effective at nucleating ice, whereas others report no ice‐nucleating ability. Here we present new experimental results for immersion mode ice nucleation by BC from two contrasting fuels (n‐decane and eugenol). We observe no significant heterogeneous nucleation by either sample. Using a global aerosol model, we quantify the maximum relative importance of BC for ice nucleation when compared with K‐feldspar and marine organic aerosol acting as INP. Based on the upper limit from our laboratory data, we show that BC contributes at least several orders of magnitude less INP than feldspar and marine organic aerosol. Representations of its atmospheric ice‐nucleating ability based on older laboratory data produce unrealistic results when compared against ambient observations of INP. Since BC is a complex material, it cannot be unambiguously ruled out as an important INP species in all locations at all times. Therefore, we use our model to estimate a range of values for the density of active sites that BC particles must have to be relevant for ice nucleation in the atmosphere. The estimated values will guide future work on BC, defining the required sensitivity of future experimental studies.

## Key Points

- Soot particles generated from two different fuels do not nucleate ice above the handling blanks when immersed in water droplets
- Previous representations of the ice‐nucleating ability of BC particles overestimate its importance in the atmosphere
- We use a global aerosol model to estimate the required ice‐nucleating efficiency of BC for it to compete with other INP in the atmosphere

## 1 Introduction

Black carbon (BC) particles emitted from both anthropogenic and natural combustion processes are ubiquitous in the present‐day atmosphere with an estimated total emission rate of 7.5 Tg/year (Bond et al., 2013). It is estimated that the anthropogenic emissions of BC have increased from ~1 Tg/year in 1850 to ~5 Tg/year in 2000 (Lee et al., 2013), which is thought to have led to a significant impact on climate (Bond et al., 2013). BC has a strong warming effect through the absorption of solar and infrared radiation, and it has been suggested that reduction in BC emissions might go some way to mitigating global warming (Bond et al., 2013). However, to accurately assess the efficacy of reducing BC emissions, it is important to quantify the impacts of BC aerosol on clouds. It is estimated that BC particles contribute substantially to global cloud condensation nuclei concentrations, and they are an important cloud condensation nuclei in industrial regions (Spracklen et al., 2011). BC therefore influences the albedo and lifetime of clouds through nucleating cloud droplets. If these immersed particles could also nucleate ice effectively, then the lifetime and albedo of supercooled clouds would be affected. This “glaciation indirect effect,” which would most likely enhance precipitation and reduce cloud lifetime, could potentially offset the aerosol effects on liquid clouds (Lohmann, 2002, 2017). However, the ice‐nucleating ability of BC under conditions pertinent to supercooled clouds remains very uncertain.

While it has been shown in laboratory studies that BC nucleates ice under conditions relevant for cirrus clouds (Hoose & Möhler, 2012; Kanji et al., 2011; Koehler et al., 2009; Kulkarni et al., 2016; Möhler, 2005; Ullrich et al., 2017), there are divergent results from laboratory and field studies of the ability of BC to nucleate ice under water‐saturated conditions, which are relevant for mixed‐phase clouds (DeMott, 1990; Diehl & Mitra, 1998; Hoose & Möhler, 2012; Schill et al., 2016; Ullrich et al., 2017). For example, a strong correlation between BC and the ice crystal concentration in a mixed‐phase orographic mountain wave cloud suggested that BC might nucleate ice (Twohy et al., 2010). However, in the same field campaign BC was not enhanced significantly in the ice crystal residues over the background air (Pratt et al., 2009). In one study of mixed‐phase clouds at a high‐altitude observatory in the Alps, soot particles only made up 5% of the submicron aerosol particles, but 27% of ice crystal residues (Cozic et al., 2008). In contrast, several studies found that BC accounted for only a minor fraction of ice crystal residues, but mineral dust was clearly enhanced (Baustian et al., 2012; Kamphus et al., 2010; Kupiszewski et al., 2016; Schmidt et al., 2017). More recently, analysis of ice‐nucleating particle (INP) chemical composition in air influenced by biomass burning events using electron microscopy showed that between 0% and 64% of INPs were BC particles and suggested that biomass burning particles could be an important regional source of INP, especially during periods when other INPs such as desert dust are absent (Mccluskey et al., 2014). Some other studies have observed an enhancement in the INP concentration in biomass burning aerosols (Levin et al., 2016; Petters et al., 2009). For cases suggesting a role of BC INPs, it is not clear if it is the BC component of these aerosols that causes nucleation or some other components mixed with it. For example, Levin et al. (2016) removed refractory BC via laser‐induced incandescence observing a decrease in the concentration of INPs for some of their samples. However, the ice‐nucleating ability of kaolinite internally mixed with BC was affected when the refractory BC part was removed by this method, which suggest that the ice‐nucleating ability of other aerosol components mixed with BC could also be affected by the removal of the refractory BC component. Measurements of the ice‐nucleating efficiencies of BC particles from diesel engines (Schill et al., 2016) found that fresh and photochemically aged BC particles did not nucleate ice effectively above their limit of detection, defined by the number of background nucleation events in their instrument. Overall, the field results do not clarify whether BC particles are consistently playing a role as INPs in the atmosphere, and they suggest that BC (or compounds generated and transported along with BC) might be playing a sporadic role in nucleating ice under certain atmospheric conditions.

The available data from laboratory studies also leave open the question of whether or not BC is an efficient INP. Some studies show evidence that BC can nucleate ice in supercooled droplets (Brooks et al., 2014; DeMott, 1990; Diehl & Mitra, 1998; Gorbunov et al., 2001; Popovicheva et al., 2008; Wright et al., 2013). Based on the available literature data (DeMott, 1990; Diehl & Mitra, 1998), a parameterization of the density of ice‐nucleating active sites (*n*_*s*_) was derived and, in combination with BC concentrations in the atmosphere, it was suggested that BC is a very important INP type (Murray et al., 2012). However, more recent studies could not reproduce similar values of *n*_*s*_ for BC, and the upper limits estimated from the limits of detection of the instruments were orders of magnitude lower (Schill et al., 2016; Ullrich et al., 2017). The large variation in BC ice nucleation activity reported in these studies means that the contribution of BC to a possible anthropogenic glaciation effect has remained poorly quantified, since modeling results will depend strongly on the represented ability of BC for nucleating ice under mixed‐phase conditions (Fan et al., 2012; Hoose, Kristjánsson, Chen, & Hazra, 2010; Hoose, Kristjánsson, & Burrows, 2010; Phillips et al., 2008, 2013; Savre & Ekman, 2015; Wang et al., 2014; Yun & Penner, 2012).

## 2 Results and Discussion

### 2.1 Laboratory Study of Ice Nucleation by Soot Samples

Given the large variation in ice‐nucleating activities reported for the relatively few experimental studies of the ice‐nucleating ability of BC, we have made new laboratory measurements. We have taken great care in these experiments to characterize the background INP, which inevitably contaminate experiments such as these, but which can lead to a false ice nucleation signal. We have also taken care to generate BC samples in a reproducible and well‐characterized way.

For these experiments, we have generated BC particles from the incomplete combustion of liquid fuels. By definition (Petzold et al., 2013), these laboratory‐generated BC samples should be referred to as soot. Much of the BC in the atmosphere originates from incomplete combustion, but on transport through the atmosphere it is expected to evolve through the adsorption of other chemical species, reactions with gas phase constituents, and aggregation with other aerosol particles; hence, this atmospheric material is then generally termed as BC. In these experiments, we consider fresh soot particles generated in our laboratory as a proxy for atmospheric BC.

**Figure 1:**
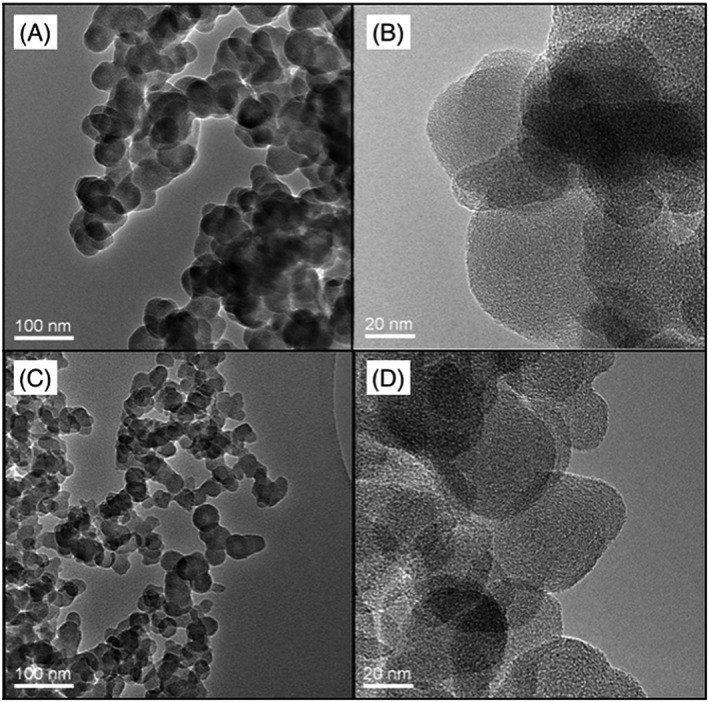
Transmission electron microscopy images of eugenol and *n*‐decane soot that were generated in the laboratory for this study. Eugenol soot images are shown on the top plates (a and b), while the plates below (c and d) are *n*‐decane soot images. The scale of each image is labeled at the bottom left‐hand corner of the image.

The fuels used to generate the soots for this study were a proxy for hydrocarbon combustion (*n*‐decane, C_10_H_22_), and a proxy for biomass burning (eugenol, C_10_H_12_O_2_). Eugenol is used as a proxy for the combustion of lignin, which constitutes 20% of pinewood (Fitzpatrick et al., 2008). Lignin has previously been shown to contribute to soot production in biomass burning, alongside cellulose (Fitzpatrick et al., 2008; J. M. Wilson et al., 2013). Consequently, soot from eugenol is similar in composition to that from pine combustion (Baeza‐Romero et al., 2010). We used the same methods to produce soot from *n*‐decane and eugenol, a wick diffusion burner with filtered air, as described in previous studies where the soot was characterized by mass spectrometry (Baeza‐Romero et al., 2010; J. M. Wilson et al., 2013; the methodology is described in detail in supporting information S1). For our experiments, soot was collected on glass slides at the top of a glass chimney and both fuels led to soot spherules with the classic fractal soot morphology (see transmission electron microscopy images, Figure 1). While they are morphologically similar, the soot from these fuels differ in several ways. For example, soot from eugenol contains larger oxygenated polyaromatic hydrocarbons, has a greater oxygen content and has a lower elemental carbon:total carbon ratio than soot from *n*‐decane (Baeza‐Romero et al., 2010; Fitzpatrick et al., 2008). Hence, we produced two contrasting examples of soot, both of which are thought to be relevant for the atmosphere.

Water suspensions of soot were prepared at 10^−3^ wt%. This is a lower mass ratio compared to similar microlitre experiments performed on mineral dusts and was selected to avoid significant aggregation of the soot, since this would lead to poor dispersion in suspension and introduce additional uncertainties into the ice nucleation measurements. Laser diffraction using a Malvern Mastersizer 2000E instrument was used to assess the particle size distributions and aggregation, the results of which are shown in the supporting information. The mastersizer records the angular intensity of scatter laser light and then estimates the particle size distribution in terms of volume equivalent spheres using Mie Theory. At 10^−1^ wt%, about 50% of the soot surface area is associated with particles >1 μm in diameter, compared with 0–20% of the surface area at the concentrations used in this study. For even more hydrophobic soots, such as acetylene burner soot, it was not possible to produce suspensions. Microlitre droplets of these suspensions were cooled down to test their ice‐nucleating efficiency. Detailed discussion of the technique can be found in Whale et al. (2014), so only a short description is presented here. Briefly, this droplet freezing assay consists of an array of droplets (between 30 and 44 droplets per experiment), pipetted on to a silanized slide (Hampton Scientific), which is cooled down at a ramp rate of −1°C/min using a stirling engine chiller. Here the active site density is used (Connolly et al., 2009), which is a singular time‐independent description of ice nucleation. For a population of *N* droplets, the fraction of frozen droplets (*ff*) is calculated as *ff*(*T*) = *n*(*T*)/*N* where *n*(*T*) is number of droplets frozen at a temperature *T*. The density of active sites *n*_*s*_ can be calculated from the fraction frozen as

(1) $$n_{s}\left(T\right)=-\frac{\ln\left(1-\mathrm{ff}\left(T\right)\right)}{A}\;$$

where *A* is the surface area of BC particles per droplet determined by Brunauer‐Emmett‐Teller (BET) multilayer adsorption. For eugenol, the specific surface area was 49.43 ± 0.89 m^2^/g, while for *n*‐decane soot it was 65.47 ± 0.81 m^2^/g.

**Figure 2:**
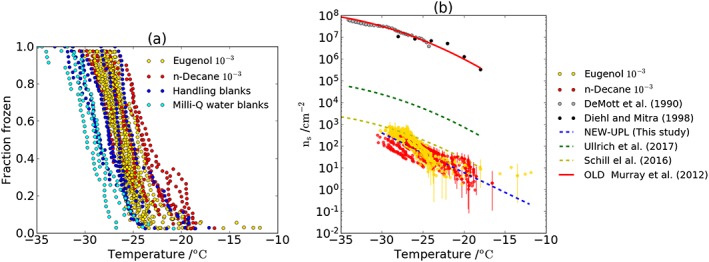
(a) Fraction frozen curves of our experiments for the two different BC samples generated from eugenol and *n*‐decane with the baseline of our experiments defined by the representative handling blanks (dark blue). The results shown are for the experiments with 10^−3^ wt%. (b) Upper limit of density of active sites that the studied BC particles can have. The errors have been estimated as the Poisson counting errors following the method presented in Harrison et al. (2016). Other parameterizations from the literature are shown for comparison. The upper limit is parameterized with the following equation: *n*_*s*_ (cm^−2^) =  exp (−6.608 − 0.419 × *T*(°C)) valid in the temperature range −30 to −12°C.

The resulting fraction of droplets frozen as a function of temperature are shown in Figure 2a for experiments with and without soot in the droplets. In all the experiments conducted, we did not measure any significant increase in ice nucleation activity above the handling blanks when soot particles were present. In these experiments the handling blanks did not freeze at the homogeneous limit but instead froze heterogeneously. This is caused by the contact between the water and the hydrophobic glass slides, or by trace contaminants. The handling blanks were MilliQ water samples run alongside the soot suspensions, reproducing every process, including sonication and stirring, that the soot suspensions were exposed to. These handling blanks froze over a broader range than standard MilliQ blanks and included freezing at warmer temperatures caused by the introduction of impurities. Given that there is no significant difference in freezing temperatures between the soot samples and handling blanks, it is not possible to ascribe the freezing temperatures measured to the influence of soot alone; instead, the freezing could either be entirely unrelated to the soot or a convolution of the soot and other contaminants acting as INPs. These results are similar to the observations of Schill et al. (2016) and Ullrich et al. (2017), where no significant ice nucleation ability of BC was observed. However, while no significant activity of these soots has been measured, they do define a limiting freezing efficiency, which we can use to draw conclusions about the potential of BC to contribute to the population of atmospheric INP.

The upper limit of the density of active sites that our soot samples can be estimated from our fraction frozen results by assuming that all the nucleation events that we observed were produced by our soot particles. The real value of *n*_s_ is likely to be lower, as the freezing events observed cannot be distinguished from the handling blanks. The density of active sites is then calculated using equation (1) with the BET‐specific surface areas of dry soot. A simple parameterization of this upper limit calculated by fitting the values to a line in logarithmic scale is given in the figure caption (see Figure 2b). The upper limit defined here (NEW‐UPL) gives smaller values of the upper limits of *n*_*s*_ than previously reported by Ullrich et al. (2017), but similar to that defined by Schill et al. (2016). All three of the recent upper limit parameterizations (this study, Schill et al., 2016; Ullrich et al., 2017) are at least 3 orders of magnitude smaller than the old parameterization defined by Murray et al. (2012) on the basis of literature data from DeMott (1990) and Diehl and Mitra (1998). DeMott (1990) used an expansion chamber to study the ice‐nucleating ability of soots generated by a acetylene burner, whereas Diehl and Mitra (1998) froze droplets containing soot particles from kerosene burner exhaust by injecting them into a wind tunnel at various temperatures.

Given the discrepancy in the observed values of *n*_*s*_ from different studies (Figure 2b), one cannot readily conclude what the typical ice‐nucleating activity of BC particles is in the atmosphere. These observed differences in BC *n*_*s*_ could be due to structural differences in various types of BC from different fuels or be related to processes affecting the BC ice‐nucleating ability such as other materials being adsorbed to the soot during production, such as organic carbon species/polyaromatic hydrocarbons. Atmospheric aging could also change the ice‐nucleating properties of BC particles as organic or sulfate coatings could potentially affect its ice‐nucleating ability (Kulkarni et al., 2016). Therefore, soot particles might have different ice‐nucleating abilities depending on their properties and sources, so assuming a single distribution of *n*_*s*_ values at all times and locations might misrepresent its ability as an INP in the atmosphere. However, an exploratory study of its atmospheric potential as INP can be done by comparing its potential contribution to global INP concentrations with that of other well‐characterized INP species.

### 2.2 Modeling the Contribution of BC INP to the Global Atmospheric Burden on INP

**Figure 3:**
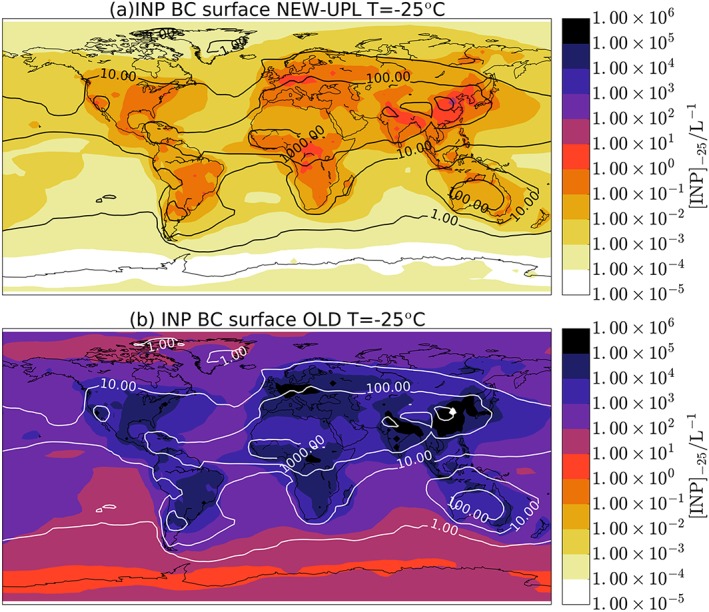
Ice‐nucleating particles (INPs) concentrations from black carbon (BC) particles and the simulated concentrations in VT17 (using feldspar and marine organic aerosols). The contour lines show [INP]_−25_ from VT17, and the color maps show the same values simulated when using BC INP calculated with (a) NEW‐UPL and (b) OLD. A similar figure for an activation temperature of −30°C is given in the supporting information.

To estimate the possible contribution of BC particles to the global distribution of INP, we use the global aerosol model GLObal Model of Aerosol Processes (GLOMAP) mode, as used in Vergara‐Temprado et al. (2017, hereafter VT17). We estimate the concentrations in the atmosphere of two well‐known ice‐nucleating aerosol species, K‐feldspar (Atkinson et al., 2013) and marine organic aerosols (T. W. Wilson et al., 2015) and compare these to the predicted contribution of BC INP. We can consider that for any aerosol species to be relevant in the atmosphere as an INP, it will have to produce similar or greater concentrations to the simulated INP concentrations of K‐feldspar and marine organics (Figure 3). Below these concentrations, it is unlikely to be an important INP; however, higher concentrations would only indicate that this is potentially more important than the two species modeled (in some locations other INP types may also be important). In our model, BC is emitted from wildfires that vary seasonally (Van der Werf et al., 2003), fossil fuel, and biofuel emissions as described in Mann et al. (2010). The annual‐mean fluxes are defined by Bond (2004). BC is emitted internally mixed with organic carbon into the insoluble Aitken mode, and then it is moved to the soluble modes by atmospheric aging. The transport, mixing, and scavenging of BC particles are driven by the meteorology of the year 2001 as used in VT17. A more detailed description of the model is given in the supporting information (see Text S2). The model simulated BC concentrations are evaluated using several data sets from the Global Aerosol Synthesis and Science Project repository presented in Reddington et al. (2017; see Text S2; Barth et al., 2015; Harris et al., 2017; Leon et al., 2016; Liu et al., 2015; Matsui et al., 2011; McMeeking et al., 2010, 2011; Metcalf et al., 2012; O'Shea et al., 2014; Oshima et al., 2012; Perring et al., 2017; Ryerson et al., 2013; Schutgens et al., 2016, 2017; Schwarz et al., 2008, 2010; Spackman et al., 2010; Subramanian et al., 2010; Taylor et al., 2014).

With the BC concentrations simulated, we can calculate the BC INP concentration for a given *n*_*s*_(*T*) following the method shown in VT17. As the reported values of *n*_*s*_ range by several orders of magnitude, we define two limiting parameterizations, one using the upper limit presented in this study (NEW‐UPL) and another using the maximum observed values reported in literature data corresponding to the parameterization shown in Murray et al. (2012; OLD; Figure 2), which was based on data from DeMott (1990) and Diehl and Mitra (1998).

**Figure 4:**
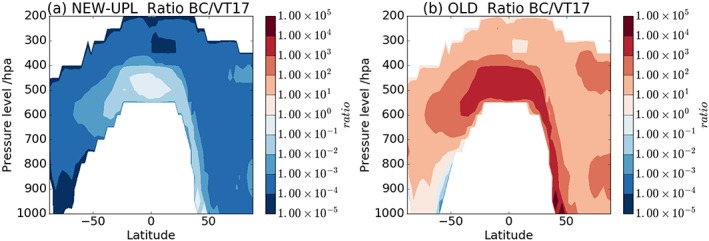
Zonal mean profiles of the ratio between the number of black carbon (BC) ice‐nucleating particles (INPs) at local ambient temperature ([INP]_ambient_) and the concentrations as simulated in VT17 for marine organics and K‐feldspar. (a) Using NEW‐UPL for calculating BC [INP]_ambient_ and (b) using OLD.

The INP distribution at the Earth's surface is shown at an activation temperature of −25°C (i.e., [INP]_−25_, where square brackets indicate concentration); that is, the number of particles that would nucleate ice if exposed to this temperature in a mixed‐phase cloud (Figure 3). These surface‐level plots are useful for assessing the distribution and makeup of the INP population around the globe, but they do not tell us where INPs can nucleate ice and influence clouds. To show this, we calculated the INP concentration throughout the atmosphere using local ambient temperatures and particle concentrations. The annual mean [INP]_ambient_ plotted in Figure 4 was calculated by averaging the daily [INP]_ambient_ values as the daily variations in temperature can substantially affect the simulated mean concentrations. At temperatures below the minimum temperature limit of each parameterization, we use the value of *n*_*s*_ for the lowest experimental temperature reported to avoid extrapolating the parameterizations.

In both the [INP]_−25_ (Figure 3) and [INP]_ambient_ plots (Figure 4), when the NEW‐UPL *n*_*s*_ values are used, the concentrations of INP from BC are several orders of magnitude smaller than simulated assuming K‐feldspar and marine organic aerosols, which suggests that BC is unlikely to play an influential role as an INP on global scales if the soot we generated is representative of atmospheric BC. However, when the OLD parameterization for *n*_*s*_ is used, BC completely dominates the global INP distribution both for [INP]_ambient_ and [INP]_−25_.

**Figure 5:**
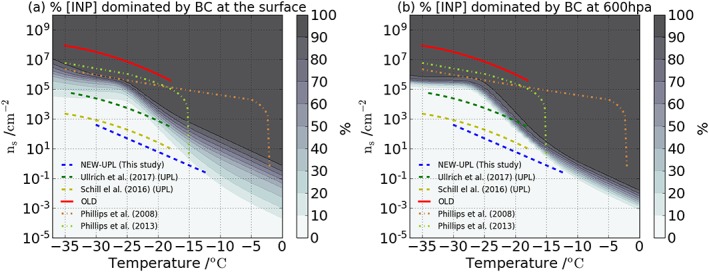
(a) Percentage of the globe surface area that would be dominated by black carbon (BC) particles at each temperature as a function of BC *n*_s_, when compared with the sum of ice‐nucleating particle (INP) produced from marine organics and K‐feldspar (from VT17). This plot is for INP at the surface. (b) Same as (a) but for a pressure level of 600 hPa. The parameterizations labeled Phillips et al. (2008) and Phillips et al. (2013) were derived using the approach given by Hoose and Möhler (2012).

Given this large difference, we estimate the *n*_s_ values required for BC to be an important INP type given present‐day BC emissions by calculating the percentage of surface grid boxes that would be dominated by BC particles if they were to have a particular *n*_*s*_ value (Figure 5) over the mixed‐phase temperature range. This is done by calculating BC [INP] from the simulated surface area distribution of BC for a range of *n*_*s*_ values (from 10^−5^ to 10^9^ cm^−2^). We then calculate at each temperature the fraction of grid boxes in our model where the BC [INP] exceeds the INP concentrations simulated by VT17, weighting each grid box by the geographic surface area that it represents. This is done for surface level aerosol concentrations (Figure 5a) and for a pressure level of 600 hPa (Figure 5b). Figure 5b predicts the range where BC would start to compete with the other species to be narrower than at surface level because of the larger homogeneity of aerosol concentrations once they are transported and mixed in the atmosphere. This approach helps us to place the other literature data for ice nucleation by BC in context and will similarly help place any future measurements of the ice nucleation ability of BC in context. The OLD parameterization and those presented by Phillips et al. (2008, 2013) fall in the range of values where BC would dominate the surface INP concentrations by orders of magnitude. On the other hand, the NEW‐UPL produces values that are orders of magnitude lower than the minimum required to influence surface concentrations. Previously reported upper limits are also lower than the required *n*_*s*_ values necessary to influence surface concentrations. We note that the model presented in VT17 is subject to low biases at high temperatures (<−15°C) in continental regions, produced probably by the absence of other terrestrial ice‐nucleating aerosols in the model, so the results at these temperatures should be interpreted accordingly.

**Figure 6:**
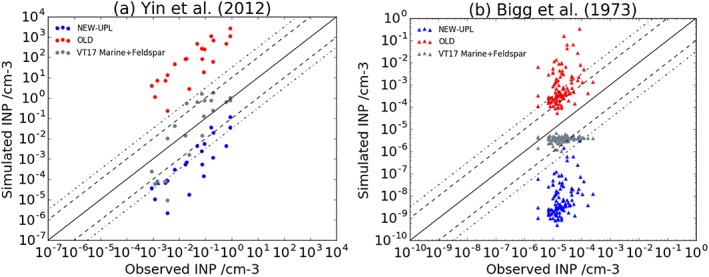
(a) Comparison between the simulated values of black carbon ice‐nucleating particles (INPs) when using NEW‐UPL and OLD parameterization, and observed INP concentrations from different places in China (Yin et al., 2012). (b) Same as (a) but for the Southern Ocean (Bigg, 1973). The comparison with the sum of marine organics and K‐feldspar (from VT17) is also shown for comparison in both panels.

We then test the realism of the OLD and NEW‐UPL parameterizations against ambient INP observations by evaluating the simulated INP concentrations against two contrasting atmospheric INP data sets, one based on remote marine locations in the Southern Ocean (Bigg, 1973) and another in a relatively higher BC loaded environment from several places in China (Yin et al., 2012; Figures 6a and 6b). The simulated concentrations of INP are calculated by applying either the NEW‐UPL or the OLD parameterization to the annual mean BC concentrations simulated at the locations where the campaigns took place. We also show for comparison the prediction with VT17. When the NEW‐UPL is used, BC alone cannot explain the observed INP concentrations and underrepresents the atmospheric concentrations by more than an order of magnitude, suggesting that other species are responsible for producing these INP concentrations. On the other hand, when the OLD parameterization is used, the measured concentrations in both environments are overestimated by more than 2 orders of magnitude, suggesting that if we consider all atmospheric BC particles to act with this efficiency in the atmosphere, we will likely overestimate their influence as INPs. This conclusion is in agreement with many field observations, which suggest that mineral dust is the dominant aerosol found in ice crystals residues (Baustian et al., 2012; Kamphus et al., 2010; Pratt et al., 2009; Schmidt et al., 2017) although others did show that BC aerosols might contribute to the observed INP concentration (Cozic et al., 2008; Mccluskey et al., 2014). K‐feldspar and marine organic aerosols can explain these atmospheric concentrations within an order of magnitude, as shown previously in Vergara‐Temprado et al. (2017). Hence, we conclude that the OLD parameterization is probably unrealistic and that it is not possible that all atmospheric BC has such a high ice‐nucleating efficiency. This conclusion has important implications for modeling studies that have previously treated BC as INP in the immersion mode (Fan et al., 2012; Hoose, Kristjánsson, Chen, & Hazra, 2010; Hoose, Kristjánsson, & Burrows, 2010; Phillips et al., 2008, 2013; Savre & Ekman, 2015; Wang et al., 2014; Yun & Penner, 2012). However, we cannot completely dismiss the potential influence that BC produced from different fuels, or exposed to different conditions, might have on INPs regionally, or during exceptional events such as large biomass burning events.

## 3 Conclusions

Our modeling estimates suggest that if all BC particles in the atmosphere behave as reported in this study, and by several other recent studies (Schill et al., 2016; Ullrich et al., 2017), BC is unlikely to play a substantial atmospheric role as INPs through the immersion mode in mixed‐phase clouds. We also conclude that a representation of BC INPs from Murray et al. (2012), which was based on the studies of DeMott (1990) and Diehl and Mitra (1998), results in an overestimation of surface‐level concentrations of INPs in remote and polluted environments by more than 2 orders of magnitude compared to observations.

The question of whether the ice‐nucleating ability of these studied BC particles is representative of the ice‐nucleating ability of atmospheric BC particles globally at all times remains open, since the discrepancies between various studies cannot be currently explained. Furthermore, we cannot rule out that atmospheric BC particles could be affected by processes enhancing their ice‐nucleating ability to levels that could make them relevant regionally or sporadically. Hence, we suggest that more studies to clarify the sources of discrepancies in the laboratory datasets are necessary to either quantify the effect of BC as INPs in the atmosphere or rule out its relevance completely. Specifically, experiments with contemporary techniques where special attention is paid to characterizing and controlling impurities need to be done with the specific BC types used in previous studies where soot was found to be an effective ice‐nucleating material. Nevertheless, we recommend that the old parameterizations, such as Murray et al. (2012), should not be used to describe the ice‐nucleating ability of all soot in the atmosphere. Overall, the available evidence suggests that BC is at most, of second‐order importance when compared to other ice‐nucleating aerosol types such as mineral dust or marine organics.

## Supporting information



Supporting Information S1
