# Supplementary material for: Is Black Carbon an Unimportant Ice‐Nucleating Particle in Mixed‐Phase Clouds?
Source: J Geophys Res Atmos. 2018 Apr 26;123(8):4273–83. doi: 10.1002/2017JD027831 (PMC6001433; doi:10.1002/2017JD027831)
Supplement: Supplementary file 1 — Supporting Information S1 [file JGRD-123-4273-s001.docx]

*Journal of Geophysical Research: Atmospheres*

Supporting Information for

Is black carbon an unimportant ice-nucleating particle in mixed-phase clouds?

Jesús Vergara-Temprado^1^*, Mark A. Holden^1,2^*, Thomas R. Orton^1,3^, Daniel O’Sullivan^1^, Nsikanabasi S. Umo^1,4^, Jo Browse^1,5^, Carly Reddington^1^, María Teresa Baeza-Romero^6^, Jenny M. Jones^7^, Amanda Lea-Langton^7,8^, Alan Williams^7^, Ken S. Carslaw^1^ and Benjamin J. Murray^1^

^1^Institute for Climate and Atmospheric Science, School of Earth and Environment, University of Leeds, Woodhouse Lane, Leeds, LS2 9JT, UK

^2^School of Chemistry, University of Leeds, Woodhouse Lane, Leeds, LS2 9JT, UK

^3^Now at: Lloyd’s of London, One Lime Street, London, EC3M 7HA, UK

^4^Now at: Institute for Meteorology and Climate Research - Atmospheric Aerosol Research, Karlsruhe Institute of Technology, Hermann-von-Helmholtz Platz 1, 76344 Eggenstein-Leopoldshafen, Germany.

^5^Now at: School of Geography, University of Exeter, Penryn Campus, Treliever Road, Penryn, Cornwall, TR10 9FE, UK

^6^Escuela de Ingeniería Industrial de Toledo, Universidad de Castilla la Mancha, Avenida Carlos III s/n, Real Fábrica de Armas, 45071 Toledo, Spain

^7^School of Chemical and Process Engineering, University of Leeds, Leeds, LS2 9JT, UK

^8^Now at: School of Mechanical, Aerospace and Civil Engineering, University of Manchester, Oxford Road, Manchester, M13 9PL, UK

Corresponding authors: J. Vergara-Temprado (eejvt@leeds.ac.uk) and M. A. Holden (m.a.holden@leeds.ac.uk)

*J. Vergara-Temprado is the corresponding author for questions related to the modelling section and M.A. Holden for the experimental part of this study. Both authors contributed equally to this work.

**Contents of this file**

Text S1 and S2

Figures S1 to S3

Table S1

***Text S1 Soot generation and freezing experiments:***

Eugenol and *n*-decane soots were generated using a diffusion burner method. The eugenol (99 %, Alfa Aesar, UK) and *n*-decane (99 %, Alfa Aesar) were placed into a metal container. A braided cotton wick, in contact with the fuel, was used to hold the flame. The length of exposed wick above the container was 5 mm. The burner was placed into a glass chimney and a 10 L min^-1^ flow of compressed air, passed through a HEPA filter, provided a source of oxygen. Once the wick was lit, the resulting soot was collected at the top of the funnel on glass microscope slides, which were removed and replaced with fresh slides every 30 seconds until the experiment was terminated.

A drop freezing assay method was used to study the ice nucleation abilities of the soot produced using the Nucleation by Immersed Particles Instrument (µL-NIPI). The method has been described previously [*Whale et al.*, 2014]. The soot particles were suspended in MilliQ ultra-pure water (18.2 MΩ.cm resistivity). To effectively disperse the soot, the suspensions were sonicated for 1-5 minutes, before being stirred with a teflon lined stirrer bar to maintain the suspension. Arrays of 1 μL droplets from each soot suspension were pipetted onto hydrophobic glass slides (siliconised glass slides, Hampton Research) that were placed on an EF600 Stirling engine cryo-cooler (Grant Asymptote). The stage was cooled at a constant ramp rate of 1 °C min^-1^, and freezing events and their corresponding temperatures were recorded using optical methods.

The soot suspension particle size distribution was measured using laser diffraction (Malvern Mastersizer 2000E). This technique is sensitive to particles with a grain size between 0.1 and 1000 μm. The suspensions were sonicated for 5 minutes before measurements. Figure S1 shows the results of laser diffraction analysis for suspensions of *n-*decane and eugenol soots of different concentrations. No significant difference in particle size distribution was observed for soot concentrations of 10^-2^ and 10^-3^ wt. %, with ≥80 % of the surface area measured associated with grain sizes below 1 μm. For soot concentrations of 10^-1^ wt. %, over 50 % of the surface area was associated with grain sizes larger than 1 μm. Nucleation significantly above the instrument baseline was not observed for any experiments. When parameterized, the results at higher concentrations will yield lower values of *n_s_* (*T*). Therefore, it is desirable to parameterize our upper limit using the highest possible concentration, since the maximum values of *n_s_* (*T*) that the soot may have can be better constrained. Since the highest concentration measurements (10^-1^ wt. %) are affected significantly by particle aggregation, the NEW-UPL parameterization was calculated using the data at 10^-3^ wt. %, which is an order of magnitude lower than the concentration at which aggregation becomes less significant. This means we are confident that no aggregation was happening in any of the samples, yet the concentration used allows enough resolution for the GLOMAP analysis, as demonstrated in Figure 4a.

***Text S2 GLOMAP modelling and evaluation:***

We use the global tropospheric model of aerosol processes (GLOMAP)[*Mann et al.*, 2010] to simulate global BC INP distribution. The model traces aerosol mass and number in 7 lognormal modes. All particles within a mode are assumed internally mixed. The model was run at 2.8° x 2.8° resolution with 31 vertical levels. The simulation was done for the year 2001 using meteorological fields diagnosed from the European Center of Medium Weather Forecast (ECMWF). Anthropogenic BC emissions come from the inventory of *Bond*, [2004] and biomass burning emisions from [*Van der Werf et al.*, 2003]. Black Carbon particles are emitted into the Aitken insoluble mode, and assumed internally mixed with co-emitted organic carbon. BC particles are aged into the soluble modes via the condensation of SO_2_ and organics whereafter they are subject to nucleation scavenging via activation in clouds.

The spatial distribution of BC mass concentration and an evaluation of the simulated BC concentrations with several aircraft campaigns using Single Particle Soot Photometer (SP2) from the GASSP database (*Reddington et al.*, [2017]) are shown in figure S2. For references for the individual datasets see Table S1.

The evaluation was done by interpolating every campaign datapoint on latitude, longitude, and height with the modelled monthly mean values. The modelled values were filtered to sizes within 90nm to 500nm to make the values comparable with the SP2 observational range. The model produces values within a factor of three from the observations for half of the analysed campaings and withing an order of magnitude from for 14 out of 16 campaigns (87.5%) (Fig 2). A larger variability is observed in the observed values than in the modelled values which is expected as we are comparing modelled values with a much lower temporal and spatial variability than the observations [*Schutgens et al.*, 2016, 2017].

BC $\left[ INP \right]_{T}$ were calculated offline using mode particle numberand BC surface area following the method shown in *Vergara-Temprado et al.*, [2017]. The method integrates numerically the lognormal size distribution assumed in GLOMAP-mode with the fraction of frozen aerosol particles at each size. The final number is obtained by adding the values from every BC containing mode. We find that the Aitken mode contributes to more than 70% of the total BC INP concentration. Using internal or external mixing assumptions among the aerosol components for calculating BC INP does not significantly change the concentrations. In order to determine the BC surface area we assume that the BC particles are spherical. BC particles in the atmosphere tend to be made up of aggregated BC spherules, typically of many 10s of nm in diameter and hence will tend to have a larger surface area than determined in our model. However, while this underestimate in surface area is highly uncertain, it is also likely to be small in magnitude. For example, a 100 nm spherical BC particle made up of an equivalent mass of 50 nm spherules will have a surface area a factor of two larger. Hence, we conclude that this underestimate is minor compared to other uncertainties in the model and measurements.

The modelling of feldspar INP builds on the modelling reported by *Vergara-Temprado et al.*, [2017] which is based on a two-species base representation of INP using K-feldspar and marine organic aerosols. The INP parameterization for K-feldspar is based on the study of *Atkinson et al.*, [2013] and the marine organic parameterization on *Wilson et al.*, [2015]


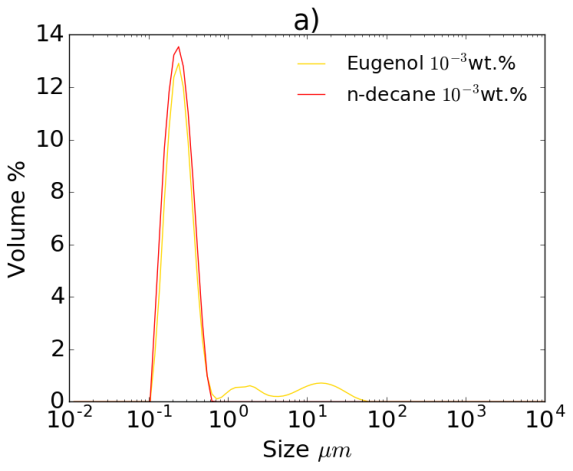

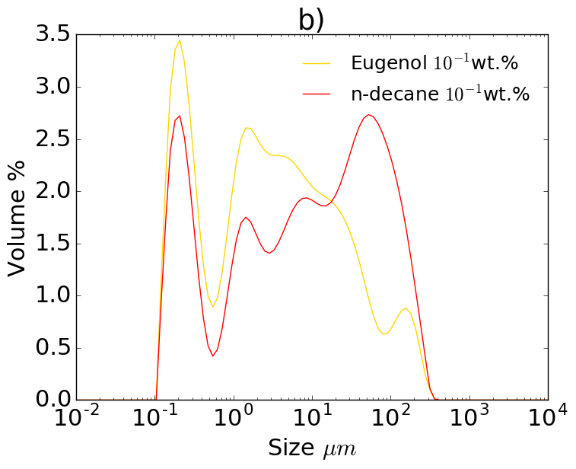


Figure S1. Plots of particle size distributions obtained for eugenol and n-decane soot suspension, at (a) 10^-3^ wt. % and (b) 10^-1^ wt. %


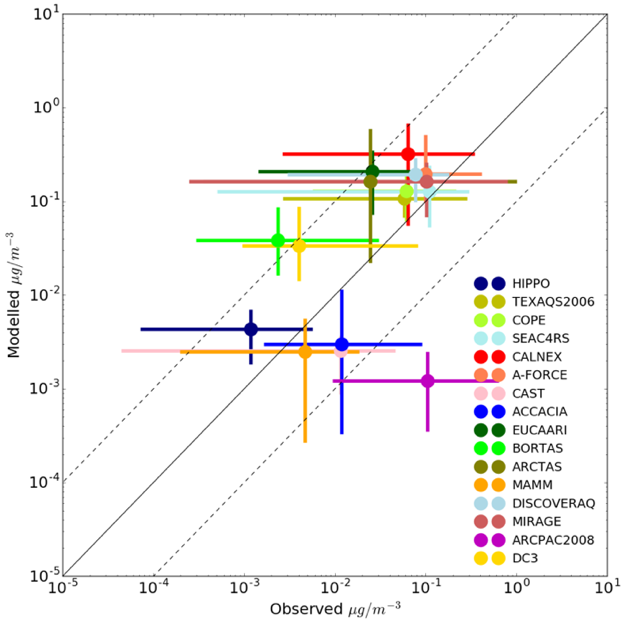


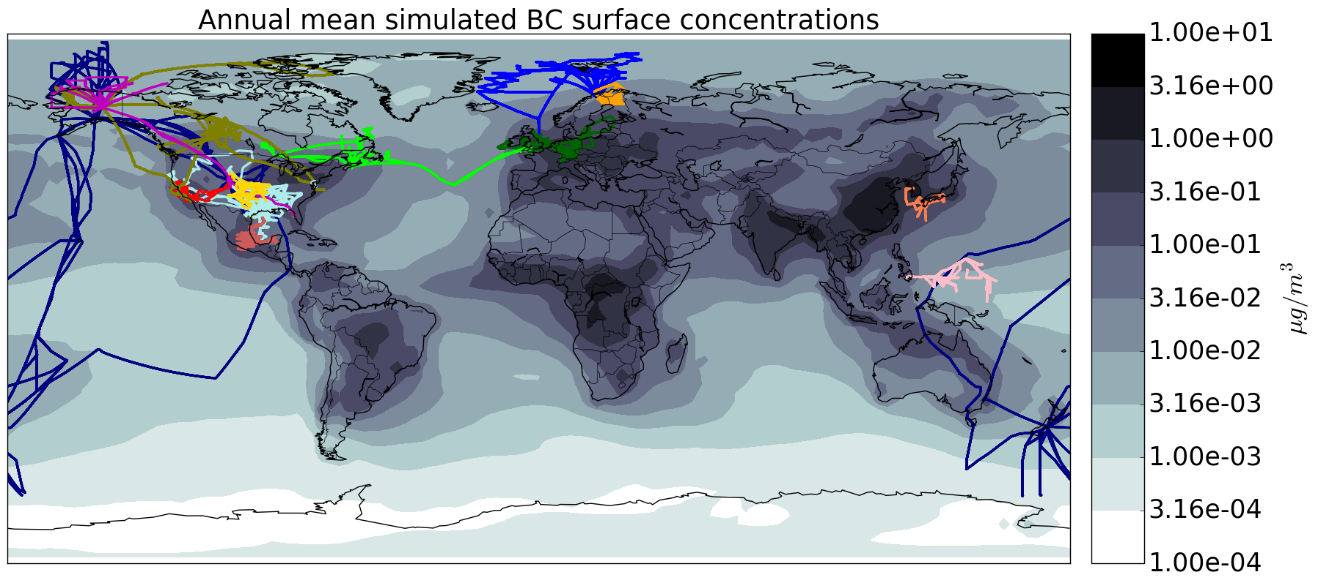


Figure S2. (Top) Annual mean surface concentration of BC mass. (Bottom) Evaluation of BC mass concentrations with several aircraft campaigns (Table S1). The campaign locations are plotted in the map following the legend in the top figure. The variability error bars represent the 95% interval of observed and modelled concentrations. The variability in the model data is lower as the comparison was done with monthly mean values for the year 2001, so it cannot capture all the temporal variability of the aircraft observations


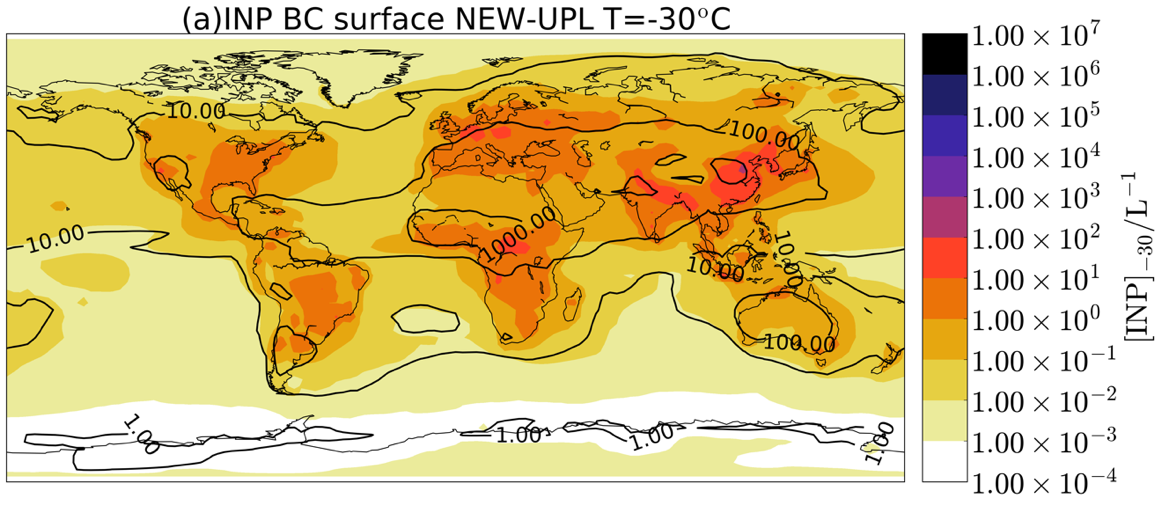

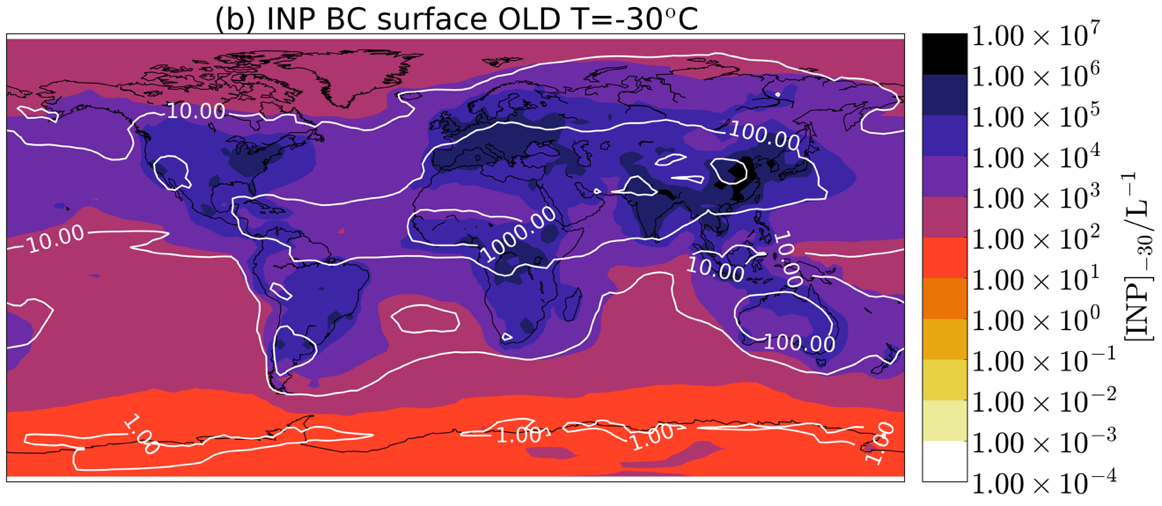


Figure S3. Similar to Figure 3 in the main text, but for an activation temperature of -30^o^ C . We note that the K-feldspar and marine organic parameterizations had to be extrapolated to reach this temperature. Also the internall/external mixing assumtions for K-feldspar aerosols start to play an important role at these temperatures, as the limiting factor to the INP concentrations starts to be the number of feldspar containing particles instead of the number of active sites.

| **Project/Campaign** | **PI(s)** | **Institute** | **Reference** |
| --- | --- | --- | --- |
| ACCACIA (Aerosol-Cloud Coupling And Climate Interactions in the Arctic) | T. W. Choularton | University of Manchester, UK | [*Liu et al.*, 2015] |
| A-FORCE (Aerosol Radiative Forcing in East Asia) | Y. Kondo | Meteorological Research Institute, Japan Meteorological Agency, Japan | [*Oshima et al.*, 2012] |
| ARCPAC-2008 (Aerosol, Radiation, and Cloud Processes affecting Arctic Climate) | J. P. Schwarz, J. R. Spackman, R.-S. Gao, , L. A. Watts, D. W. Fahey | NOAA Earth System Research Laboratory (ESRL) Chemical Sciences Division, USA | [*Spackman et al.*, 2010] |
| ARCTAS (Arctic Research of the Composition of the Troposphere from Aircraft and Satellites) | Y. Kondo | Meteorological Research Institute, Japan Meteorological Agency, Japan | [*Matsui et al.*, 2011] |
| BORTAS (Boreal forest fires on Tropospheric oxidants over the Atlantic using Aircraft and Satellite) | H. Coe, J. Allan | University of Manchester, UK | [*Taylor et al.*, 2014] |
| CALNEX (California Research at the Nexus of Air Quality and Climate Change) | J. P. Schwarz, J. R. Spackman, R.-S. Gao, , L. A. Watts, D. W. Fahey | NOAA ESRL Chemical Sciences Division, USA | [*Metcalf et al.*, 2012] |
| CAST (Co-ordinated Airborne Studies in the Tropics) | M. Gallagher | University of Manchester, UK | [*Harris et al.*, 2017] |
| COPE (the Convective Precipitation Experiment) | T. W. Choularton | University of Manchester, UK | [*Leon et al.*, 2016] |
| DC3 (Deep Convective Clouds & Chemistry) | B. Weinzierl | Deutsches Zentrum für Luft- und Raumfahrt, Institute of Atmospheric Physics, Germany | [*Barth et al.*, 2015] |
| DISCOVER-AQ (Deriving Information on Surface Conditions from Column and Vertically Resolved Observations Relevant to Air Quality) | B. E. Anderson | NASA Langley Research Center, USA | [*Ryerson et al.*, 2013] |
| EUCAARI-LONGREX (European Integrated Project on Aerosol Cloud Climate and Air Quality Interactions – Long-range Experiment) | H. Coe | University of Manchester, UK | [*McMeeking et al.*, 2010, 2011] |
| HIPPO (HIAPER Pole-to-Pole Observations) | J. P. Schwarz | NOAA ESRL Chemical Sciences Division, USA | [*Schwarz et al.*, 2010] |
| MAMM (Methane and other greenhouse gases in the Arctic – Measurements, process studies and modelling) | H. Coe | University of Manchester, UK | [*O’Shea et al.*, 2014] |
| MIRAGE (Megacities Impact on Regional and Global Environment) | A. Clarke, G. Kok | University of Hawaii, USA; Droplet Measurement Technologies, USA | [*Subramanian et al.*, 2010] |
| SEAC4RS (Studies of Emissions and Atmospheric Composition, Clouds and Climate Coupling by Regional Surveys) | R.-S. Gao, J. P. Schwarz, A.E. Perring, M. Markovic, J. Holloway | NOAA ESRL Chemical Sciences Division, USA | [*Perring et al.*, 2017] |
| TEXAQS- 2006 (Texas Air Quality Study) | J. P. Schwarz, J. R. Spackman, R.-S. Gao, , L. A. Watts, D. W. Fahey | NOAA ESRL Chemical Sciences Division, USA | [*Schwarz et al.*, 2008] |

Table S1. Information on the Single Particle Soot Photometer (SP2) datasets obtained from the GASSP database (*Reddington et al.*, [2017]]) and used for model evaluation (Section S2).

**References:**

Atkinson, J. D., B. J. Murray, M. T. Woodhouse, T. F. Whale, K. J. Baustian, K. S. Carslaw, S. Dobbie, D. O’Sullivan, and T. L. Malkin (2013), The importance of feldspar for ice nucleation by mineral dust in mixed-phase clouds., *Nature*, *498*(7454), 355–8, doi:10.1038/nature12278.

Barth, M. C. et al. (2015), The deep convective clouds and chemistry (DC3) field campaign, *Bull. Am. Meteorol. Soc.*, *96*(8), 1281–1310, doi:10.1175/BAMS-D-13-00290.1.

Bond, T. C. (2004), A technology-based global inventory of black and organic carbon emissions from combustion, *J. Geophys. Res.*, *109*(D14), D14203, doi:10.1029/2003JD003697.

Harris, N. R. P. et al. (2017), Coordinated airborne studies in the tropics (CAST), *Bull. Am. Meteorol. Soc.*, *98*(1), 145–162, doi:10.1175/BAMS-D-14-00290.1.

Leon, D. C. et al. (2016), The Convective Precipitation Experiment (COPE): Investigating the origins of heavy precipitation in the southwestern United Kingdom, *Bull. Am. Meteorol. Soc.*, *97*(6), 1003–1020, doi:10.1175/BAMS-D-14-00157.1.

Liu, D. et al. (2015), The importance of Asia as a source of black carbon to the European Arctic during springtime 2013, *Atmos. Chem. Phys.*, *15*(20), 11537–11555, doi:10.5194/acp-15-11537-2015.

Mann, G. W., K. S. Carslaw, D. V. Spracklen, D. a. Ridley, P. T. Manktelow, M. P. Chipperfield, S. J. Pickering, and C. E. Johnson (2010), Description and evaluation of GLOMAP-mode: a modal global aerosol microphysics model for the UKCA composition-climate model, *Geosci. Model Dev.*, *3*(2), 519–551, doi:10.5194/gmd-3-519-2010.

Matsui, H. et al. (2011), Seasonal variation of the transport of black carbon aerosol from the Asian continent to the Arctic during the ARCTAS aircraft campaign, *J. Geophys. Res. Atmos.*, *116*(5), 1–19, doi:10.1029/2010JD015067.

McMeeking, G. R. et al. (2010), Black carbon measurements in the boundary layer over western and northern Europe, *Atmos. Chem. Phys.*, *10*(19), 9393–9414, doi:10.5194/acp-10-9393-2010.

McMeeking, G. R., W. T. Morgan, M. Flynn, E. J. Highwood, K. Turnbull, J. Haywood, and H. Coe (2011), Black carbon aerosol mixing state, organic aerosols and aerosol optical properties over the United Kingdom, *Atmos. Chem. Phys.*, *11*(17), 9037–9052, doi:10.5194/acp-11-9037-2011.

Metcalf, A. R., J. S. Craven, J. J. Ensberg, J. Brioude, W. Angevine, A. Sorooshian, H. T. Duong, H. H. Jonsson, R. C. Flagan, and J. H. Seinfeld (2012), Black carbon aerosol over the Los Angeles Basin during CalNex, *J. Geophys. Res. Atmos.*, *117*(8), 1–24, doi:10.1029/2011JD017255.

O’Shea, S. J. et al. (2014), Methane and carbon dioxide fluxes and their regional scalability for the European Arctic wetlands during the MAMM project in summer 2012, *Atmos. Chem. Phys.*, *14*(23), 13159–13174, doi:10.5194/acp-14-13159-2014.

Oshima, N. et al. (2012), Wet removal of black carbon in Asian outflow: Aerosol Radiative Forcing in East Asia (A-FORCE) aircraft campaign, *J. Geophys. Res. Atmos.*, *117*(3), 1–24, doi:10.1029/2011JD016552.

Perring, A. E. et al. (2017), In situ measurements of water uptake by black carbon-containing aerosol in wildfire plumes, *J. Geophys. Res. Atmos.*, *122*(2), 1086–1097, doi:10.1002/2016JD025688.

Reddington, C. L. et al. (2017), THE GLOBAL AEROSOL SYNTHESIS AND SCIENCE PROJECT (GASSP): Measurements and modelling to reduce uncertainty, *Bull. Am. Meteorol. Soc.*, BAMS-D-15-00317.1, doi:10.1175/BAMS-D-15-00317.1.

Ryerson, T. B. et al. (2013), The 2010 California Research at the Nexus of Air Quality and Climate Change (CalNex) field study, *J. Geophys. Res. Atmos.*, *118*(11), 5830–5866, doi:10.1002/jgrd.50331.

Schutgens, N., S. Tsyro, E. Gryspeerdt, D. Goto, N. Weigum, M. Schulz, and P. Stier (2017), On the spatio-temporal representativeness of observations, *Atmos. Chem. Phys.*, *17*(16), 9761–9780, doi:10.5194/acp-17-9761-2017.

Schutgens, N. A. J., E. Gryspeerdt, N. Weigum, S. Tsyro, D. Goto, M. Schulz, and P. Stier (2016), Will a perfect model agree with perfect observations? The impact of spatial sampling, *Atmos. Chem. Phys.*, *16*(10), 6335–6353, doi:10.5194/acp-16-6335-2016.

Schwarz, J. P. et al. (2008), Measurement of the mixing state, mass, and optical size of individual black carbon particles in urban and biomass burning emissions, *Geophys. Res. Lett.*, *35*(13), 1–5, doi:10.1029/2008GL033968.

Schwarz, J. P., J. R. Spackman, R. S. Gao, L. A. Watts, P. Stier, M. Schulz, S. M. Davis, S. C. Wofsy, and D. W. Fahey (2010), Global-scale black carbon profiles observed in the remote atmosphere and compared to models, *Geophys. Res. Lett.*, *37*(18), 1–5, doi:10.1029/2010GL044372.

Spackman, J. R., R. S. Gao, W. D. Neff, J. P. Schwarz, L. A. Watts, D. W. Fahey, J. S. Holloway, T. B. Ryerson, J. Peischl, and C. A. Brock (2010), Aircraft observations of enhancement and depletion of black carbon mass in the springtime Arctic, *Atmos. Chem. Phys.*, *10*(19), 9667–9680, doi:10.5194/acp-10-9667-2010.

Subramanian, R. et al. (2010), Black carbon over Mexico: the effect of atmospheric transport on mixing state, mass absorption cross-section, and BC/CO ratios, *Atmos. Chem. Phys.*, *10*(1), 219–237, doi:10.5194/acp-10-219-2010.

Taylor, J. W. et al. (2014), Size-dependent wet removal of black carbon in Canadian biomass burning plumes, *Atmos. Chem. Phys.*, *14*(24), 13755–13771, doi:10.5194/acp-14-13755-2014.

Vergara-Temprado, J. et al. (2017), Contribution of feldspar and marine organic aerosols to global ice nucleating particle concentrations, *Atmos. Chem. Phys.*, *17*(5), 3637–3658, doi:10.5194/acp-17-3637-2017.

Van der Werf, G. R., J. T. Randerson, G. J. Collatz, and L. Giglio (2003), Carbon emissions from fires in tropical and subtropical ecosystems, *Glob. Chang. Biol.*, *9*(4), 547–562, doi:10.1046/j.1365-2486.2003.00604.x.

Whale, T. F., B. J. Murray, D. O’Sullivan, N. S. Umo, K. J. Baustian, J. D. Atkinson, and G. J. Morris (2014), A technique for quantifying heterogeneous ice nucleation in microlitre supercooled water droplets, *Atmos. Meas. Tech. Discuss.*, *7*(9), 9509–9536, doi:10.5194/amtd-7-9509-2014.

Wilson, T. W. et al. (2015), A marine biogenic source of atmospheric ice-nucleating particles, *Nature*, *525*(7568), 234–238, doi:10.1038/nature14986.
